# Identification and Evaluation of Autoantibody to a Novel Tumor-Associated Antigen GNA11 as a Biomarker in Esophageal Squamous Cell Carcinoma

**DOI:** 10.3389/fonc.2021.661043

**Published:** 2021-09-10

**Authors:** Huimin Wang, Xiaoang Yang, Guiying Sun, Qian Yang, Chi Cui, Xiao Wang, Hua Ye, Liping Dai, Jianxiang Shi, Jianying Zhang, Peng Wang

**Affiliations:** ^1^Henan Institute of Medical and Pharmaceutical Sciences in Academy of Medical Science, Zhengzhou University, Zhengzhou, China; ^2^School of Basic Medical Sciences, Zhengzhou University, Zhengzhou, China; ^3^Henan Key Laboratory of Tumor Epidemiology, Zhengzhou University, Zhengzhou, China; ^4^State Key Laboratory of Esophageal Cancer Prevention & Treatment, Zhengzhou University, Zhengzhou, China; ^5^College of Public Health, Zhengzhou University, Zhengzhou, China

**Keywords:** GNA11, tumor-associated antigen, autoantibody, immunodiagnosis, esophageal squamous cell carcinoma

## Abstract

The study aims to explore the diagnostic value of anti-GNA11 autoantibody in esophageal squamous cell carcinoma (ESCC) from multiple levels. Autoantibody against GNA11 with the highest diagnostic performance was screened out from the customized protein microarray. A total of 486 subjects including ESCC patients and matched normal controls were recruited in the verification and validation phases by using enzyme-linked immunosorbent assay (ELISA). Western blotting analysis was used to verify the ELISA results. Immunohistochemistry (IHC) was used to evaluate GNA11 expression in ESCC tissues and para-tumor tissues. In addition, a bioinformatics approach was adopted to investigate the mRNA expression of GNA11 in ESCC. Results indicated that the level of anti-GNA11 autoantibody in ESCC patients was significantly higher than that in the normal controls, and it can be used to distinguish ESCC patients from normal individuals in clinical subgroups (*p* < 0.05), as revealed by both ELISA and Western blotting. The receiver operating characteristic (ROC) curve analysis showed that anti-GNA11 autoantibody could distinguish ESCC patients from normal controls with an area under the ROC curve (AUC) of 0.653, sensitivity of 10.96%, and specificity of 98.63% in the verification cohort and with an AUC of 0.751, sensitivity of 38.24%, and specificity of 88.82% in the validation cohort. IHC manifested that the expression of GNA11 can differentiate ESCC tissues with para-tumor tissues (*p* < 0.05), but it cannot be used to differentiate different pathological grades and clinical stages (*p* > 0.05). The mRNA expression of GNA11 in ESCC patients and normal controls was different with a bioinformatics mining with The Cancer Genome Atlas (TCGA) and Genotype-Tissue Expression (GTEx) data in Gene Expression Profiling Interactive Analysis (GEPIA). In summary, anti-GNA11 autoantibody has the potential to be a new serological marker in the diagnosis of ESCC.

## Introduction

Esophageal cancer (EC) is one of the common malignant tumors that threaten the health of human beings, with its incidence ranking seventh and cancer-related death ranking sixth worldwide (1). Studies estimated that there were 572,000 new cases and 508,600 deaths of EC around the world in 2018 (2). The incidence of EC in China is among the top 5 in the world (1). It was estimated that there were 246,000 new cases and 188,000 deaths of EC in 2015, which was the fourth leading cause of cancer-related death in China (3). Esophageal squamous cell carcinoma (ESCC) accounts for more than 90% of EC in China, which is a severe healthcare burden (4).

The 5-year overall survival rate of EC is less than 20%, and the prognosis is inferior (5, 6). The poor prognosis of EC is mainly due to the lack of clinical symptoms in patients in the early stage and the paucity of reliable non-invasive testing methods. However, relevant studies pointed out that early diagnosis of EC can significantly improve its poor prognosis, with a 5-year survival rate reaching as high as 80%–90% (7, 8). The standard diagnostic methods for EC screening include endoscopy and pathological biopsy, which are expensive and invasive. Consequently, a critically unmet need in the diagnosis and management of EC is identifying and developing novel non-invasive biomarkers that can complement the traditional diagnostic methods. Researchers have suggested that autoantibodies against tumor-associated antigens (TAAs) could be used as diagnostic biomarkers for the early diagnosis of cancer. These anti-TAA autoantibodies are stable in the circulating blood and can be produced as early as several years before the appearance of clinical symptoms (9–12). Currently, there are a lot of autoantibodies against TAAs that have been used in the early diagnosis of ESCC, such as FOXP3 (13), Fascin (14), Ezrin (15), STIP1 (16), LY6K (17), and MMP7 (18). However, the sensitivity and specificity of these anti-TAA autoantibodies cannot meet the needs of the clinical diagnosis of ESCC as biomarkers. Hence, it is of great importance to identify additional biomarkers with high specificity and sensitivity for the diagnosis of ESCC.

**Figure 1:**
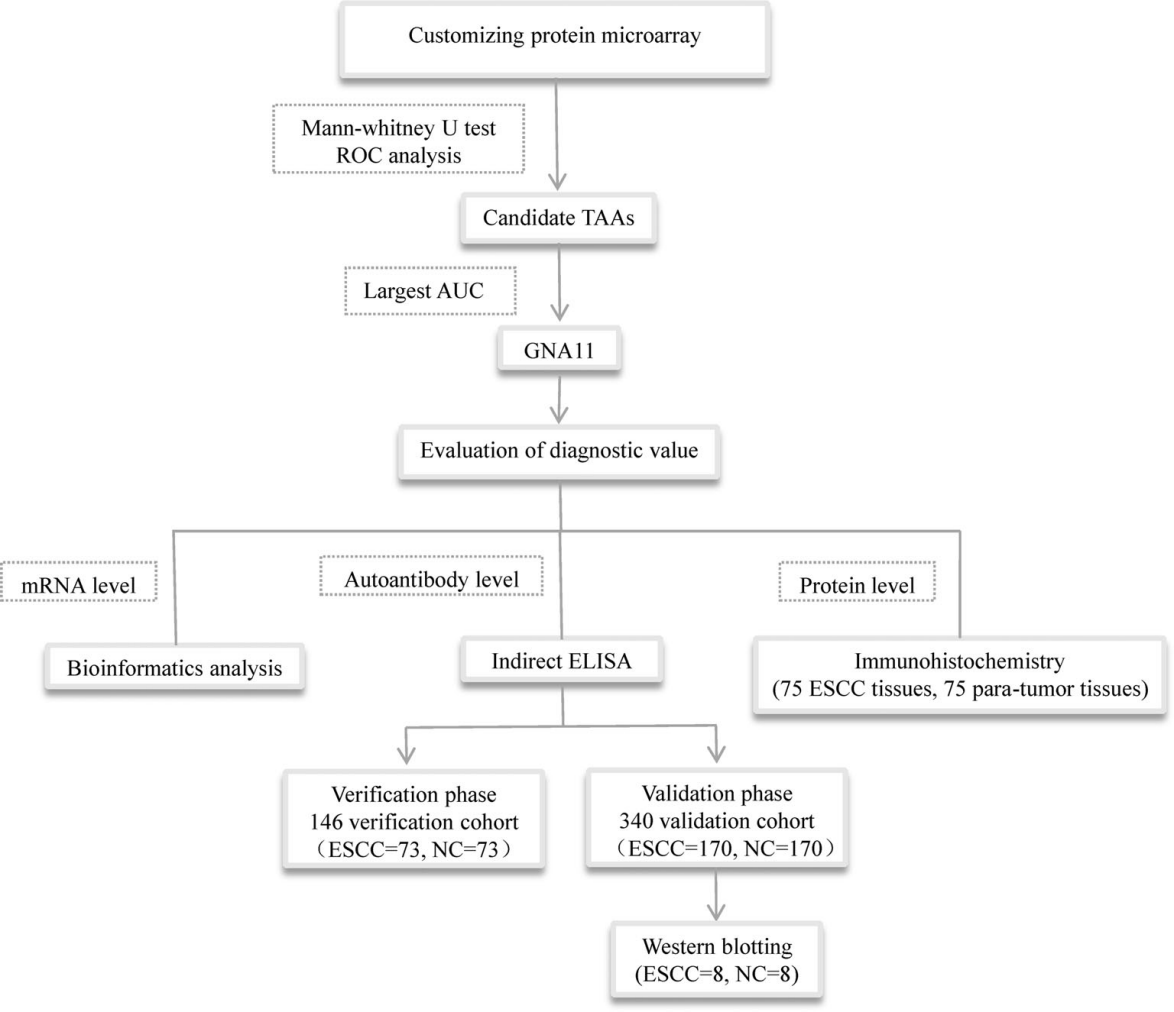
The overall design of the study. TAAs, tumor-associated antigens; ELISA, enzyme-linked immunosorbent assay.

Heterotrimeric G proteins are widely expressed in all eukaryotic cells consisting of three subunits: alpha, beta, and gamma. GNA11 is the alpha subunit of G protein and is involved in a variety of transmembrane signaling systems. Our previous study indicated that GNA11 was involved in the development of hepatocellular carcinoma (HCC) and might be a potential biomarker in HCC detection (19). Our previous study also found that anti-GNA11 autoantibody had the potential to diagnose ESCC when we established a diagnostic model of an autoantibody panel to screen ESCC (20). *GNA11* gene was frequently mutated in the conventional esophageal adenocarcinoma, and the mutation was related to critical cellular pathways including PI3K, RAS, and MAPK, which suggested that GNA11 mutation might be tightly linked to the occurrence of EC (21). In the current study, in order to evaluate the potential of anti-GNA11 autoantibody in the diagnosis of ESCC, the level of anti-GNA11 autoantibody in sera of ESCC patients and matched normal controls was detected by ELISA, and the protein level and mRNA level of GNA11 were further explored by immunohistochemistry (IHC) and bioinformatics. The overall design of the current study is shown in **Figure 1**.

## Materials and Methods

### Serum Samples

Serum samples of 333 ESCC patients and 293 normal controls were used in the study. From July 2017 to October 2018, sera from ESCC patients were collected from a third-level grade A hospital in Henan Province, China. Normal control sera were derived from the biological specimen bank in Henan Key Laboratory of Tumor Epidemiology. All patients underwent pathological examination to confirm that they have not received any treatment prior to collecting blood, and none of the controls had autoimmune diseases or tumor-related diseases. Sera from 90 ESCC patients and 50 normal controls were selected for protein microarray assay, and 486 sera from 243 ESCC patients and 243 normal controls were used for ELISA. **Table 1** shows the detailed clinical information of all participants. The peripheral blood of all subjects under fasting state was collected at 5 ml and placed into vacuum tube without anticoagulants. The collected whole blood samples were centrifuged at 3,000 rpm for 10 min after standing for 1 h at room temperature and then stored at −80°C. This study has been approved by the Ethics Committee of Zhengzhou University, and all the subjects had signed informed consent.

**Table 1:** Characteristics of study participants.

Variables	Verification phase (n = 146)		Validation phase (n = 340)	
	ESCC	NC	ESCC	NC
Number	73	73	170	170
Gender				
Male, n (%)	46 (63.01)	46 (63.01)	117 (68.82)	117 (68.82)
Female, n (%)	27 (36.99)	27 (36.99)	53 (31.18)	53 (31.18)
Mean age ± SD (years)	63.8 ± 9.1	63.6 ± 9.0	63.8 ± 8.1	64.8 ± 8.3
Age range (years)	41–82	41–82	42–88	45–88
Tumor site				
Upper thorax	9 (12.33)		25 (14.71)	
Middle thorax	39 (53.42)		51 (30.00)	
Lower thorax	24 (32.88)		37 (21.76)	
Unknown	1 (1.37)		57 (33.53)	
Family tumor history				
Yes	22 (30.14)		27 (15.9)	
No	49 (67.12)		45 (26.5)	
Unknown	2 (2.74)		98 (57.6)	
Histological grade				
High	2 (2.74)		4 (2.35)	
Medium	23 (31.51)		48 (28.24)	
Low	22 (30.14)		35 (20.59)	
Unknown	26 (35.61)		83 (48.82)	
TNM stage				
I	14 (19.18)		60 (35.29)	
II	17 (23.29)		21 (12.35)	
III	28 (38.36)		7 (4.12)	
IV	8 (10.96)		4 (2.35)	
Unknown	6 (8.21)		78 (45.89)	
Lymph node metastasis				
Positive	42 (57.53)		25 (14.71)	
Negative	27 (36.99)		51 (30.00)	
Unknown	4 (5.48)		94 (55.29)	
Distant metastasis				
Yes	8 (10.96)		3 (1.76)	
No	60 (82.19)		62 (36.47)	
Unknown	5 (6.85)		105 (61.77)	

ESCC, esophageal squamous cell carcinoma; NC, normal control.

### Protein Microarray

The study was authorized by Guangzhou Bochong Biotechnology Co., Ltd., to make focused array protein microarray including 154 recombinant human proteins or protein fragments (CDI lab), which included 143 proteins or protein fragments encoded by 138 cancer driver genes and 11 proteins with high diagnostic value in our previous studies containing IMP1, IMP2, IMP3, CyclinB1, c-Myc, CIP2A/p90, RalA, YWHAZ, RBM39, and two fragments of Survivin. Protein microarray was used to detect the expression levels of corresponding autoantibodies in 140 serums to screen out significant TAAbs associated with ESCC. To eliminate the bias brought by the difference of background values, signal-to-noise ratio (SNR) was defined as F median/B median, where F532 Median refers to the median of the foreground value of signal points in the 532-nm channel and B532 Median refers to the median of the background value of signal points in the 532-nm channel. The detailed experimental procedure was performed exactly as the standard protocol described in our previous study (22).

### Enzyme-Linked Immunosorbent Assay

The level of anti-GNA11 autoantibody in sera of ESCC patients and normal controls was detected by ELISA. Purified recombinant protein GNA11 was purchased from the LD Biopharma Company (San Diego, USA). Horseradish peroxidase (HRP)-conjugated mouse anti-human IgG (Wuhan Aoko Biotechnology Co. Ltd.) was used as the secondary antibody. Each ELISA plate included eight standard concentrations of 10, 20, 50, 100, 150, 200, 250, and 300 ng/ml of IgG (Solarbio); a positive control; a negative control; and a blank control, which enabled the stability of all the plates. The detailed operation of ELISA was described in our previous study (23). The optical density (OD) of each well was measured at 450 and 620 nm by using a microplate reader (Thermo Fisher Scientific).

### Western Blotting

The positive and negative sera of anti-GNA11 autoantibody found by ELISA were randomly selected and detected by Western blotting to verify the immunoreactivity of the sera. The detailed procedure of Western blotting was described in our previous study (24). Briefly, purified recombinant protein GNA11 was electrophoresed by 10% sodium dodecyl sulfate–polyacrylamide gel electrophoresis (SDS-PAGE) gel and then transferred onto nitrocellulose membrane. Selected serum samples diluted at 1:100 and HRP-conjugated mouse anti-human IgG antibody with a dilution of 1:5,000 were utilized as the primary antibody and the secondary antibody, respectively. The positive reaction signal was obtained by Azure Biosystems with chemiluminescence (C300–C600) according to the manufacturer’s instructions.

### Immunohistochemistry

Immunohistochemical analysis was performed to detect the expression level of GNA11 protein in 75 ESCC tissues and 75 corresponding para-tumor tissues. Tissue microarray (TMA; CGT No. HEso-Squ150CS-02) and the specific operations were provided by Shanghai Outdo Biotech Co. Ltd. Mouse monoclonal anti-GNA11 antibody was used as the first antibody. Biotin-labeled secondary antibody and diaminobenzidine (DAB) were used as detecting reagents. All the TMA results were evaluated by two independent pathologists without knowing the tumor stage of the TMA sections. Ten fields were randomly selected under the microscope for each microarray, and the score was calculated according to the percentage of positive cells (area score) and staining intensity (color score). There were five scoring conditions for the percentage of stained cells in a cell count, for example, score 0 (less than 10%), 1 (10%–25%), 2 (26%–50%), 3 (51%–75%), and 4 (more than 75%). Staining intensity incorporated four different evaluation criteria: negative, score 0; faint yellow, score 1; brown yellow, score 2; and medium brown, score 3. The final score was attained by multiplying the score of the percentage of positive cells by the staining intensity score, which was negative score 0, weakly positive score 1–4, positive score 5–8, and strongly positive score 9–12.

### Bioinformatics Analysis

The expression of GNA11 at the mRNA level was further explored in Gene Expression Profiling Interactive Analysis (GEPIA) (http://gepia.cancer-pku.cn/). The Cancer Genome Atlas (TCGA) and Genotype-Tissue Expression (GTEx) normal data were chosen to be compared in esophageal carcinoma (ESCA) patients about the difference of GNA11 expression at the mRNA level. RNA sequencing expression data of ESCC were according to TCGA dataset. And the use of GEPIA was precisely based on the report from literature (25).

### Statistical Analysis

IBM statistical software (version 25.0) and GraphPad Prism 8.0 were utilized in the study. All statistical analysis processes were two-tailed tests, and the test level was set as α = 0.05. The non-parametric test was applied to compare the difference of anti-GNA11 autoantibody between ESCC patients and normal controls. The frequency of anti-GNA11 autoantibody in different clinical parameters and demographic characteristics in all ESCC patients was analyzed by chi-square test. One-way ANOVA was used to analyze IHC scores in different tissue types. The receiver operating characteristic (ROC) curve was performed to estimate the diagnostic ability of the biomarker in different groups and clinical subgroups. At the same time, Youden’s index, positive likelihood ratio (PLR), negative likelihood ratio (NLR), positive predictive value (PPV), negative predictive value (NPV), and accuracy were also calculated to appraise the better ability of anti-GNA11 autoantibody in differentiating ESCC patients from normal controls. The area under the ROC curve (AUC) values of different clinical subgroups were analyzed by DeLong test. The cutoff value was set as mean value plus one SD of the normal control OD values (mean ± SD).

## Results

### Autoantibody to a Novel Tumor-Associated Antigen Was Found in Sera of Esophageal Squamous Cell Carcinoma Patients by Protein Microarray Technology

**Figure 2:**
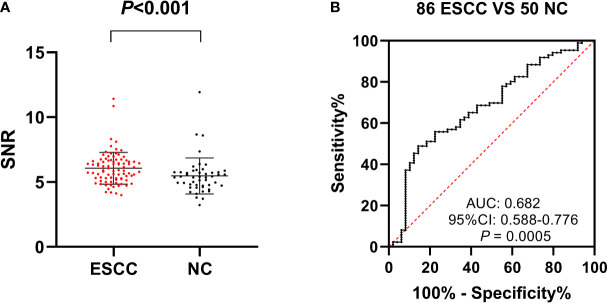
The expression level and diagnostic value of anti-GNA11 autoantibody in ESCC patients and normal controls in screening phase. **(A)** The scatter plot depicts the level of anti-GNA11 autoantibody in ESCC group and normal control group. **(B)** The receiver operating characteristic curve of anti-GNA11 autoantibody in the screening stage. ESCC, esophageal squamous cell carcinoma; NC, normal controls; SNR, signal-to-noise ratio.

In the present study, 86 sera from ESCC patients and 50 control sera were finally selected to evaluate the autoantibody levels with protein microarray. Interestingly, five candidate anti-TAA autoantibodies corresponding to P53, PTEN, GNA11, GNAS, and SRSF2 were identified by the Mann–Whitney U test and ROC analysis (20). In the ESCC group and normal control group, the positive rates of five anti-TAA autoantibodies ranged from 16.28% to 34.88% and 8.16% to 16.33%, respectively. The AUC values of five autoantibodies in diagnosing ESCC ranged from 0.606 to 0.682. The AUC value of anti-GNA11 autoantibody was the highest (0.682, 95% CI: 0.588–0.776) (**Figure 2B**) with sensitivity of 17.44% and specificity of 91.84%. The positive rate of anti-GNA11 autoantibody in the ESCC group and normal control group was 17.44% and 8.16%, respectively. The scatter plot showed that the level of anti-GNA11 autoantibody in the ESCC group was obviously higher than that in the normal control group (*p* < 0.05) (**Figure 2A**). It indicated that anti-GNA11 autoantibody has the potential to distinguish ESCC patients from normal controls. To verify the hypothesis, the performance of anti-GNA11 autoantibody was further explored in cohorts with a large sample size.

### Validating Diagnostic Value of Anti-GNA11 Autoantibody in Sera of Esophageal Squamous Cell Carcinoma Patients and Normal Controls

**Figure 3:**
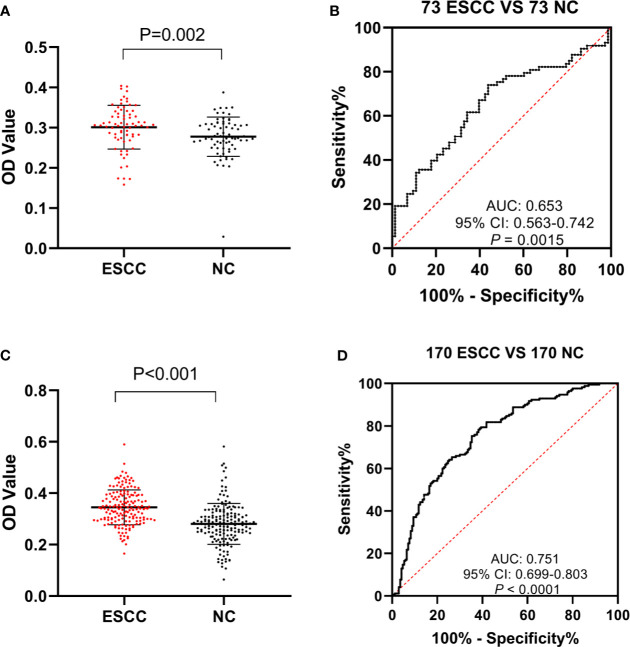
The expression level and diagnostic value of anti-GNA11 autoantibody in the phases of verification and validation. **(A, C)** The expression level of anti-GNA11 autoantibody in ESCC patients and normal controls in verification cohort and validation phase. **(B, D)** ROC analysis of anti-GNA11 autoantibody in the verification and validation phase. ESCC, esophageal squamous cell carcinoma; NC, normal controls; ROC, receiver operating characteristic.

To further validate the efficacy of anti-GNA11 autoantibody in the immunodiagnosis of ESCC, sera from 243 ESCC patients and 243 normal controls were used in ELISA. All 486 serum samples were divided into two cohorts according to the ratio of 3:7. In the verification cohort (73 ESCC *vs*. 73 NC), a higher expression level of anti-GNA11 autoantibody was observed in ESCC patients (mean ± SD: 0.301 ± 0.054) compared with normal controls (mean ± SD: 0.277 ± 0.049) (**Figure 3A**); and anti-GNA11 autoantibody can distinguish 10.96% of ESCC patients with an AUC of 0.653 at the specificity of 98.63% (**Figure 3B**). A validation cohort of larger sample size (170 ESCC *vs*. 170 NC) was used to further confirm the diagnostic value of anti-GNA11 autoantibody. In this validation cohort, the level of anti-GNA11 autoantibody was distinctly higher in the ESCC group (mean ± SD: 0.345 ± 0.068) than that in the normal control group (mean ± SD: 0.281 ± 0.079) (**Figure 3C**), and the AUC value was as high as 0.751 with sensitivity of 38.24% and specificity of 88.82% (**Figure 3D**). The detailed results are shown in **Table 2**.

**Table 2:** The diagnostic value of anti-GNA11 autoantibody in two stages of validation.

Cohorts	AUC	95% CI	Se (%)	Sp (%)	YI	+LR	−LR	PPV (%)	NPV (%)	Accuracy (%)
Verification	0.653	0.563–0.742	10.96	98.63	9.59	8.00	0.90	88.89	52.55	0.55
Validation	0.751	0.699–0.803	38.24	88.82	27.06	3.42	0.70	77.38	58.98	0.64

AUC, area under the curve; CI, confidence interval; Se, sensitivity; Sp, specificity; YI, Youden’s index; +LR, positive likelihood ratio; −LR, negative likelihood ratio; PPV, positive predictive value; NPV, negative predictive value.

**Figure 4:**
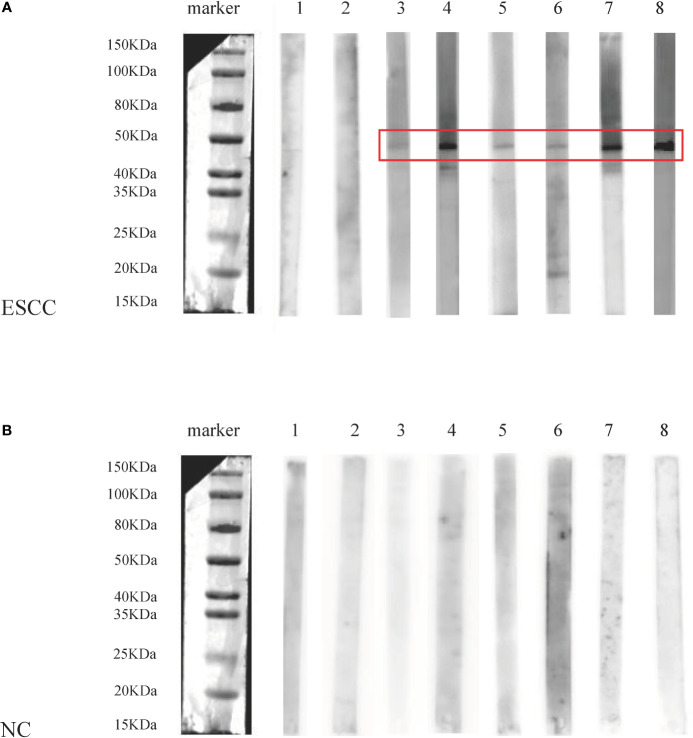
The analysis of anti-GNA11 autoantibody by Western blotting in sera of eight ESCC patients and eight normal controls. ESCC, esophageal squamous cell carcinoma; NC, normal controls. **(A)** The results of Western blotting in sera of 8 ESCC patients, of which No. 1-2 was negative, and No. 3-8 showed a positive band near 45 kDa. **(B)** The results of Western blotting in 8 control sera were negative, and there was no positive band near 45 kDa.

Additionally, eight ESCC and eight normal control sera were randomly selected from ESCC patients and normal controls for Western blotting analysis to further confirm the results of ELISA. The molecular weight of GNA11 protein is 45.8 kDa. Results indicated that six of eight ESCC sera showed positive bands with molecular weight near 45 kDa, and none of the eight control sera showed a positive band in the corresponding 45 kDa position. The results of Western blotting are shown in **Figure 4** and were consistent with those of ELISA.

### The Effect of Anti-GNA11 Autoantibody in Different Clinicopathologic Characteristics of Esophageal Squamous Cell Carcinoma

**Figure 5:**
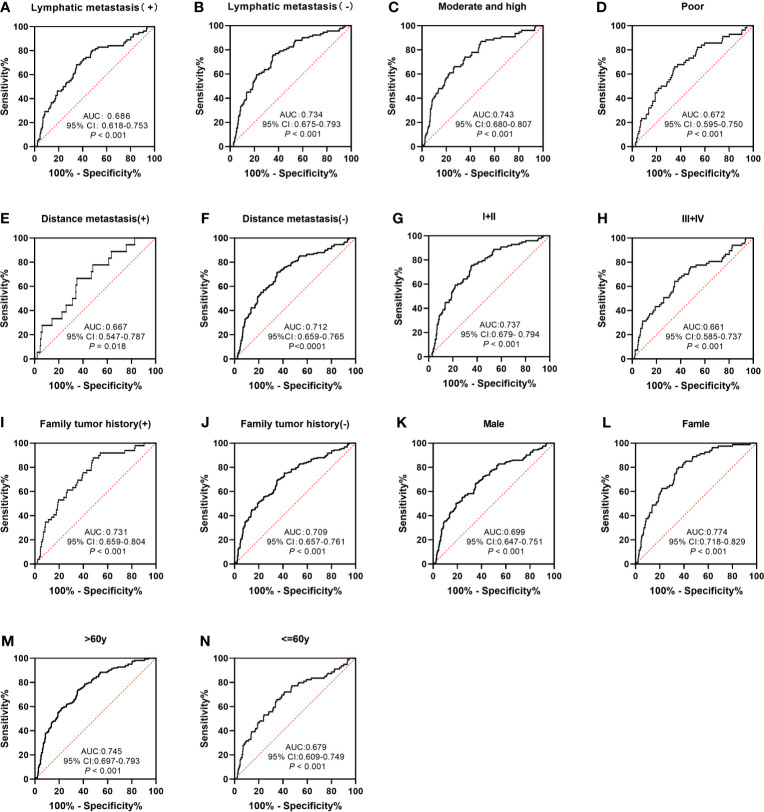
The performance of anti-GNA11 autoantibody according to different clinical variables of ESCC patients. ESCC, esophageal squamous cell carcinoma.

The performance of anti-GNA11 autoantibody in clinical variables (including lymphatic metastasis, differentiation, distance metastasis, TNM stage, family tumor history, gender, and age) was further explored. Anti-GNA11 autoantibody could significantly distinguish ESCC patients from normal controls in every subgroup (*p* < 0.05) (**Figures 5A–N**). The AUC values of clinical subgroups ranged from 0.661 to 0.774 (*p* < 0.05). The maximum AUC of 0.774 was observed among female ESCC patients (**Figure 5L**), and the minimum AUC of 0.661 was detected in ESCC patients with TNM stage III and IV (**Figure 5H**). The AUC values in comparison groups regarding lymphatic metastasis, differentiation, distance metastasis, TNM stage, family tumor history, and age was not significantly different (*p* > 0.05), yet it presented a marginal difference in gender (*p* = 0.05). In addition, there were no differences in the positive rates of anti-GNA11 autoantibody in all subgroups (*p* > 0.05) (**Table 3**).

**Table 3:** The positive frequencies of anti-GNA11 autoantibody in subgroups.

Categories	Number	Frequency (%)	*p*
Lymphatic metastasis			
Positive	82	26.83	0.468
Negative	91	31.87	
Differentiation			
Moderate and high	77	38.96	0.055
Low	56	23.21	
Distance metastasis			
Positive	18	27.78	0.744
Negative	149	31.54	
Family tumor history			
Positive	49	30.61	0.703
Negative	158	33.54	
Age range (years)			
>60	164	35.37	0.441
≤60	79	30.38	
Gender			
Male	163	32.52	0.563
Female	80	36.25	
TNM stage			
I–II	98	32.65	0.558
III–IV	67	28.36	

### The Expression Level of mRNA and Protein of GNA11

**Figure 6:**
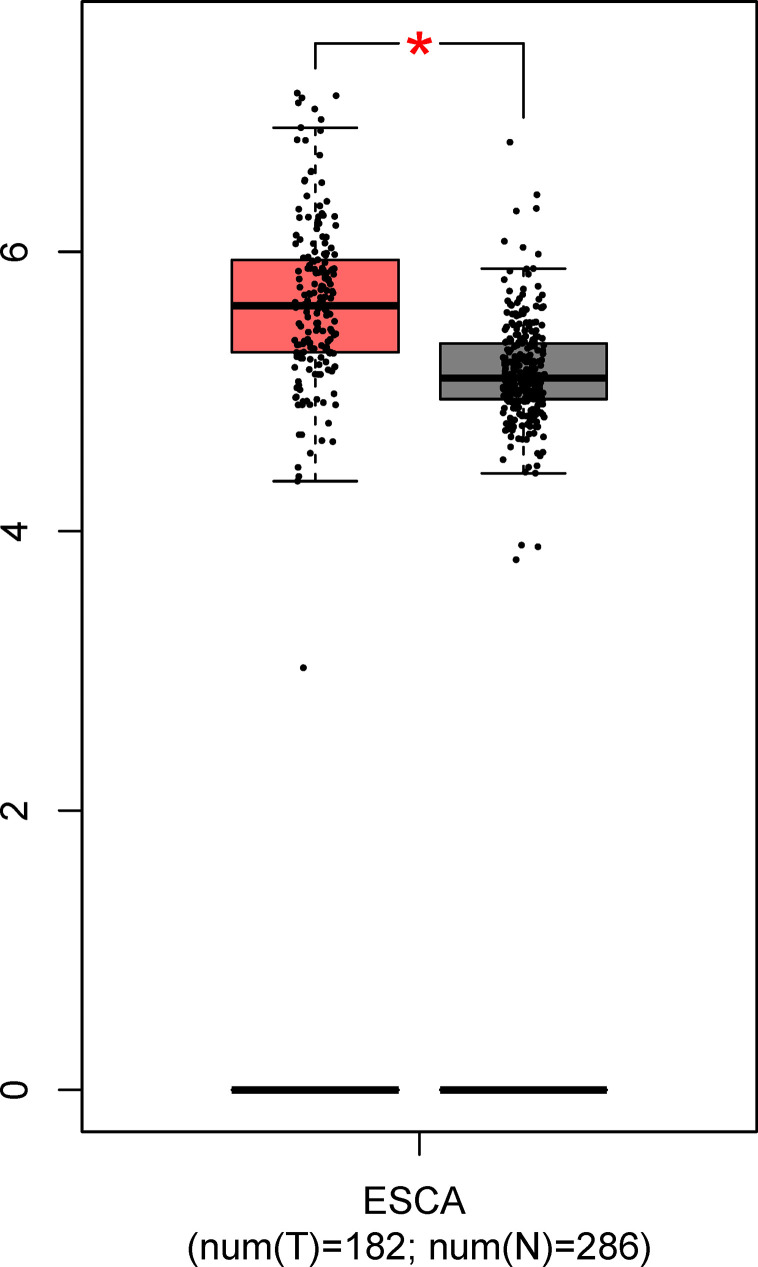
The expression of GNA11 at mRNA level based on TCGA and GTEx data in GEPIA. The red box represents the esophageal carcinoma group, and the gray box chart indicates the normal control group. ESCA, esophageal carcinoma; TCGA, The Cancer Genome Atlas; GTEx, Genotype-Tissue Expression; GEPIA, Gene Expression Profiling Interactive Analysis. ^*^ *p* < 0.05.

As shown in **Figure 6**, the mRNA level of GNA11 was higher (fold change > 1.4, *p* < 0.05) in ESCC patients compared with normal controls based on TCGA and GTEx data in GEPIA, which was consistent with the difference in serum level of anti-GNA11 autoantibody between ESCC patients and normal controls.

Furthermore, the expression of GNA11 at the protein level was investigated in the Human Protein Altas (HPA; https://www.proteinatlas.org/). However, there was no prior evidence on the expression of GNA11 at protein in ESCC tissues (**Supplementary Figure 1**). Therefore, IHC was employed to further verify the expression of GNA11 at the protein level in ESCC tissues and para-tumor tissues. TMA slides including 75 ESCC tissue samples and corresponding para-tumor tissues were commercially acquired for this study. The clinical information of 75 ESCC patients including American Joint Committee on Cancer (AJCC) clinical stage and pathological grade was analyzed to explore the association between GNA11 expression and tumor stage. The expression level of GNA11 in tumor tissues and para-tumor tissues was evaluated by staining intensity (color score) and the percentage of positive cells (area score).

**Figure 7:**
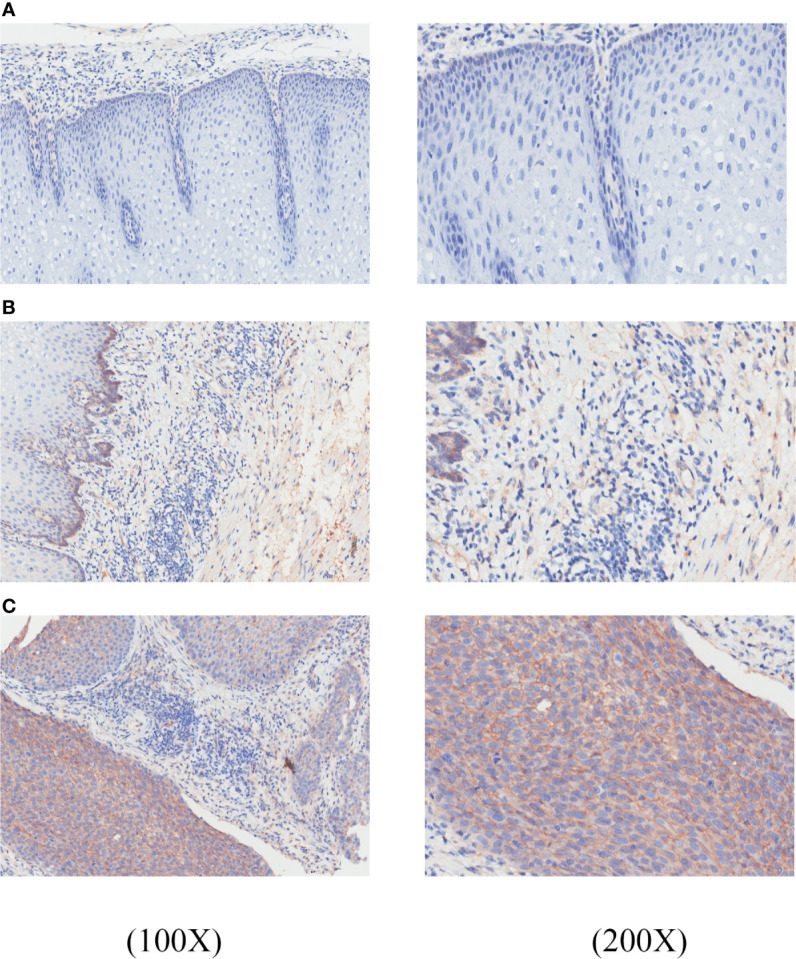
GNA11 is overexpressed in ESCC tissues compared with corresponding para-tumor tissues by immunohistochemistry. The histopathology of these tissue samples was tested by hematoxylin and eosin (H&E) staining. **(A)** A representative negative expression of GNA11 protein in para-tumor tissues at ×100 and ×200 magnification. **(B)** Weak positive expression of GNA11 protein in representative para-tumor tissues at ×100 and ×200 magnification. **(C)** Positive expression of GNA11 protein in representative ESCC tissues at ×100 and ×200 magnification. ESCC, esophageal squamous cell carcinoma.

Results showed that GNA11 was overexpressed in ESCC tissues compared with para-tumor tissues (final score 1.57 *vs*. final score 0.14, *p* < 0.05) (**Table 4**). The color scores in ESCC tissues were significantly higher than those in para-tumor tissues, whereas the area scores were similar in different groups. In addition, the expression of GNA11 protein was distinctly higher in ESCC patients of pathological grade III compared with pathological grade I and II patients in color score (*p* < 0.05). The area score showed boundary difference in ESCC patients of clinical stage 1–2 compared with clinical stage 3–4 (*p* = 0.05). And the difference in final score was only observed in ESCC patients of pathological grade I compared with pathological grade III (*p* < 0.05). The color score and final score in ESCC patients with clinical stage 3–4 were also higher than those in clinical stage 1–2 patients (*p* < 0.05). **Figure 7** includes representative immunostaining for GNA11 protein in ESCC tissues and corresponding para-tumor tissues.

**Table 4:** The expression of GNA11 between ESCC tissues and para-tumor tissues evaluated by IHC.

Tissue types	N	Color				Area				Final score			
		Score	*p* ^a^	*p* ^b^		Score	*p* ^a^	*p* ^b^		Score	*p* ^a^	*p* ^b^	
Tumor	75	1.99	0.00			0.79	0.71			1.57	0.00		
Pathological grade													
I	17	1.47	0.01	0.01^c^	0.62^d^	0.65	1.00	0.15^c^	0.66^d^	0.96	0.04	0.02^c^	0.45^d^
II	34	1.71	0.00	0.01^c^		0.74	0.98	0.22^c^		1.27	0.00	0.06^c^	
III	24	2.75	0.00			0.96	0.24			2.64	0.00		
Clinical stage													
1–2	34	1.5	0.00	0.02^e^		0.62	0.98	0.05^e^		0.93	0.00	0.01^e^	
3–4	41	2.39	0.00			0.93	0.13			2.22	0.00		
Para-tumor	75	0.21				0.67				0.14			

p^a^: compared with para-tumor tissues (one-way ANOVA). p^b^: Comparisons between two groups. For pathological grades, c means comparisons of ESCC patients with pathological grade I and II versus pathological grade III; d means comparisons of pathological grade I ESCC patients with pathological grade II. For clinical stages, e represents comparisons of clinical stage 1–2 ESCC patients with clinical stage 3–4. The final score is obtained by multiplying area and color. The data shown in the table are the mean values of the samples. ESCC, esophageal squamous cell carcinoma; IHC, immunohistochemistry.

## Discussion

Currently, the discovery of reliable biomarkers is still an important goal for the diagnosis of ESCC. However, only a few biomarkers had been used in the clinical detection of ESCC due to the limitation in study population or diagnostic value. So far, only a few tumor-associated proteins have been approved to be used as biomarkers in cancer (26). Therefore, it is necessary to find autoantibodies to TAAs as effective and reliable biomarkers. Protein microarray is an emerging high-throughput technique for protein detection and analysis with the merits of low sample consumption, simultaneous detection of multiple proteins, automation, and high sensitivity (27, 28). It has been widely used to detect protein biomarkers in a variety of cancers such as lung cancer, gastric cancer, and colorectal cancer (29–31). In the current study, the protein microarray containing 154 recombinant proteins was customized to screen out potential TAAs as biomarkers for the diagnosis of ESCC by using ROC analysis and Wilcoxon test. Through an extensive screening and validation, anti-GNA11 autoantibody was finally identified as a potential biomarker for the diagnosis of ESCC. Our study demonstrated that the positive rate of anti-GNA11 autoantibody was 38.24% (65/170), which was significantly higher than that in sera of normal controls 11.18% (19/170) (*p* < 0.05). Besides, our team further explored the performance of anti-GNA11 autoantibody as a biomarker in different subgroups including lymphatic metastasis, differentiation, distance metastasis, family tumor history, TNM stage, gender, and age. However, there was no obvious difference to distinguish these subgroups. Similar results had been seen in other studies about ESCC detection. It was reported that autoantibodies to p53, NY-ESO-1, matrix metalloproteinase-7 (MMP-7), heat shock protein 70 (Hsp70), and peroxiredoxin VI (Prx VI) had no difference in the detection of early-stage and late-stage ESCC patients (32). Chen et al. indicated that tumor-associated autoantibodies against Fascin also showed no difference in histological grade or TNM stage (14). It can be inferred that anti-GNA11 autoantibody may have a relation to the occurrence of ESCC rather than the progression from results above. Through bioinformatics analysis, our team also found that the mRNA level of GNA11 in ESCC is significantly higher than that in the normal control group. IHC analysis further confirmed that GNA11 protein was overall more abundant in ESCC tissues compared with para-tumor tissues. Multiple expression level analyses showed that anti-GNA11 autoantibody is elevated in ESCC patients.

*GNA11* involves signaling pathways associated with cell survival including PI3K, RAS, and MAPK; and mutations of the corresponding genes could give the cancer cells a selective growth advantage that develop in a restricted nutritional condition (33). Mutations in *GNA11* will lead to malformations and overgrowth of capillary in capillary malformation patients (34). Some studies have demonstrated that *GNAQ/11* mutation occurs at the early stage of uveal melanoma and clonal expansion is possible only after *GNAQ/11* mutation happens in normal cells (35, 36). Another study indicated that hotspot mutations of Gαq and Gα11 (R183 and Q209) that occurred in uveal melanoma acting as cancer driver genes will destroy the function of GTPase in the MAPK/MEK/ERK pathway, and the implementation of *GNAQ/GNA11* mutation analysis in clinical diagnosis processes might be helpful in making treatment decisions (37). GNA* (*GNAS*, *GNAQ*, or *GNA11*) aberrations in gastrointestinal excluding appendiceal and colorectal cancer account for 7.3% in all GNA* mutated tumors, and there is a trend toward lower median overall survival in patients with GNA* mutated tumors compared with GNA* wild-type tumors (38). Gα11 is coupled to gonadotropin-releasing hormone receptor (GnRHR) and also involved in negatively regulating the growth of human breast epithelial cells through GnRH–GnRHR system (39). Studies suggested that the methylation of *GNA11* resulted in reduced mRNA expression in breast cancer, which was of great help in the growth of human breast cancer cells (40). It was reported that 80% uveal melanomas have *GNAQ* or *GNA11* mutations, which were potential drivers of MAPK activation; and a randomized phase II trial of selumetinib, a selective MEK inhibitor, has shown prospective therapeutic effect for uveal melanomas (41, 42). Consistent with the above studies, we also detected elevated levels of anti-GNA11 autoantibody in sera of ESCC patients, which may indicate changes in *GNA11* gene during the original initiation of ESCC.

The present study reports the potential value of anti-GNA11 autoantibody in the diagnosis of ESCC patients from multiple levels. Anti-GNA11 autoantibody was initially screened out by protein microarray, and the performance of the autoantibody was further verified in two stages to obtain stable and reliable results. From multiple level analyses, we systematically confirmed that anti-GNA11 autoantibody is elevated in ESCC patients and may be a novel marker for the diagnosis of ESCC patients. However, some other studies on *GNA11* mainly focus on the influence of its mutation on melanoma, and there are few studies working on *GNA11* in ESCC. We believe that our subsequent studies on the mechanism of *GNA11* in the occurrence and development of ESCC will fill this gap and provide more data to support our current findings.

## Conclusions

In summary, this study has investigated the expression of GNA11 in ESCC from multiple levels. The results have demonstrated that anti-GNA11 autoantibody can be used as a potential biomarker in the detection of ESCC patients.

## Data Availability Statement

The raw data supporting the conclusions of this article will be made available by the authors, without undue reservation.

## Ethics Statement

The studies involving human participants were reviewed and approved by the Institution Review Board of Zhengzhou University. The patients/participants provided their written informed consent to participate in this study. Written informed consent was obtained from the individual(s) for the publication of any potentially identifiable images or data included in this article.

## Author Contributions

PW, XY, and JZ conceived the design of the current study. HW and GS conducted experiments and drafted the manuscript. QY and CC participated in the data analysis. HY, XW, JS, and LD helped to draft the manuscript. All authors contributed to the article and approved the submitted version.

## Funding

This work was supported by The Funded Project of International Training of High-level talents in Henan Province, Zhengzhou Major Project for Collaborative Innovation (18XTZX12007) and the Major Project of Science and Technology in Henan Province (161100311400).

## Conflict of Interest

The authors declare that the research was conducted in the absence of any commercial or financial relationships that could be construed as a potential conflict of interest.

## Publisher’s Note

All claims expressed in this article are solely those of the authors and do not necessarily represent those of their affiliated organizations, or those of the publisher, the editors and the reviewers. Any product that may be evaluated in this article, or claim that may be made by its manufacturer, is not guaranteed or endorsed by the publisher.

## Supplementary Material

Supplementary Figure 1 The expression of GNA11 protein in the tissues of different organs.
