# Supplementary figures and images for: Identification and Evaluation of Autoantibody to a Novel Tumor-Associated Antigen GNA11 as a Biomarker in Esophageal Squamous Cell Carcinoma
Source: Front Oncol. 2021 Sep 10;11:661043. doi: 10.3389/fonc.2021.661043 (PMC8462091; doi:10.3389/fonc.2021.661043)

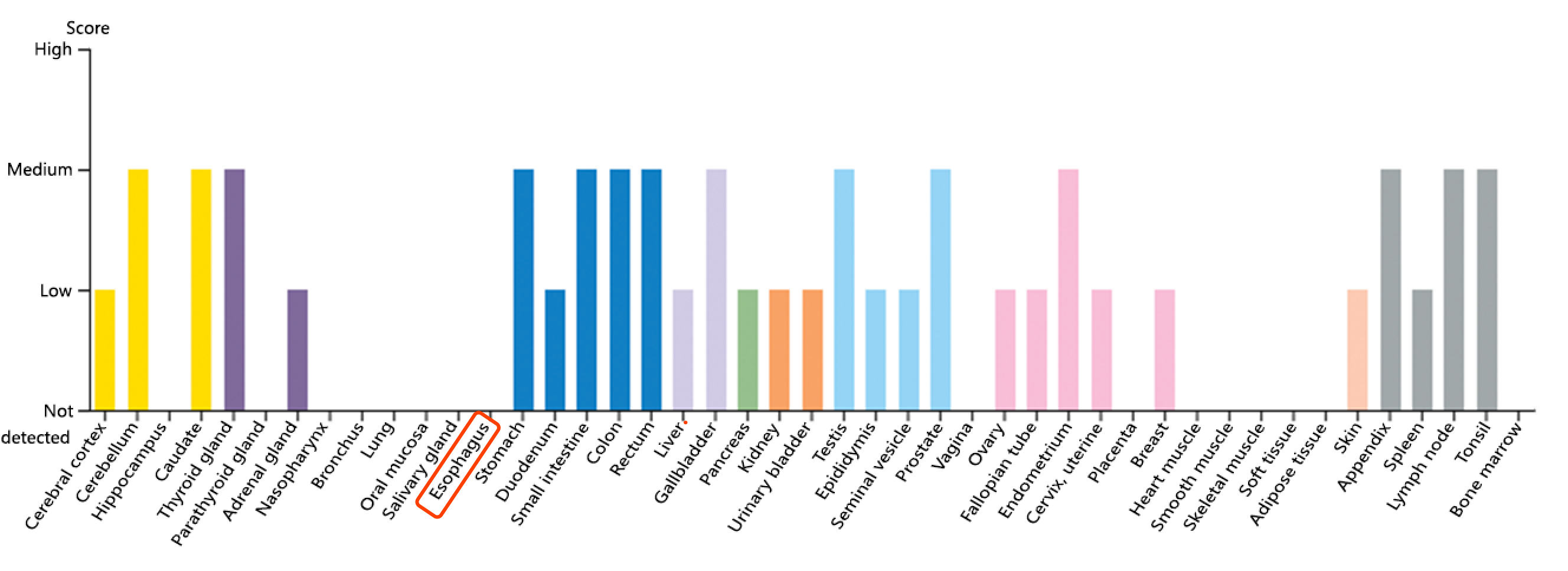

Supplement: Supplementary Figure 1 — The expression of GNA11 protein in the tissues of different organs. [file Image_1.tif]
